# Preparing a scientific manuscript in Linux: Today's possibilities and limitations

**DOI:** 10.1186/1756-0500-4-434

**Published:** 2011-10-22

**Authors:** Vakhtang Tchantchaleishvili, Jan D Schmitto

**Affiliations:** 1 Department of Surgery, University of Minnesota, Minneapolis, Minnesota, USA; 2 Division of Cardiothoracic, Transplantation and Vascular Surgery, Hannover Medical School, University of Hannover, Hannover, Germany

**Keywords:** Linux, operating system, manuscript, scientific software

## Abstract

**Background:**

Increasing number of scientists are enthusiastic about using free, open source software for their research purposes. Authors' specific goal was to examine whether a Linux-based operating system with open source software packages would allow to prepare a submission-ready scientific manuscript without the need to use the proprietary software.

**Findings:**

Preparation and editing of scientific manuscripts is possible using Linux and open source software. This letter to the editor describes key steps for preparation of a publication-ready scientific manuscript in a Linux-based operating system, as well as discusses the necessary software components. This manuscript was created using Linux and open source programs for Linux.

## Introduction

Today's science is experiencing an increasingly merciless competition between the proprietary and open concepts. Free, open access scientific literature is not merely a proven concept, but its popularity is increasing dramatically. Each year we are witnessing a significant growth of free, open source computer software. It is becoming more widely accepted in science, pushing the limits of free science further and further, to previously unimaginable horizons.

Increasing number of scientists are now enthusiastic about using free, open source software for their research purposes [1]. From this standpoint, a question emerges if it possible to use a free, open-source operating system as a platform to create scientific work in a free, open source environment? Although preparing and editing scientific manuscripts is only a small part of this tremendous work, it still requires a variety of computer software, and is obviously related to financial costs. Authors' specific goal was to examine if a Linux-based operating system with its free, open source software packages would suffice to prepare a submission-ready manuscript, without the need to use a proprietary computer software.

## Linux-based operating systems

Linux, in simplest terms, is a most widely used free, open source operating system [2]. Linux-based operating systems are used on various types of hardware ranging form servers (Google [3], Wikipedia [4]) to cell phones (Android [5]). Most of the world's supercomputers run Linux [6]. For use in personal computers, Linux is packaged as part of a various distributions which, in addition to the operating system, also includes desktop environment and a large collection of software applications. The result is a fully functional, free, open source operating system natively supporting various types of software - a necessity for preparing and editing scientific manuscripts in a free, open source environment.

Most popular Linux distributions include Ubuntu, Debian, Fedora, etc.[7]. Notably, there is a dedicated Linux distribution for scientific laboratories called Scientific Linux (Scientific Linux, Fermi National Accelerator Laboratory, Batavia, Illinois, and the European Organization for Nuclear Research - CERN, Geneva, Switzerland), packaged with various free, open source scientific software [8]. Modern-day Linux-based operating systems for desktop computers and laptops are very powerful and stable, fully functional [9], suitable for scientific work [10].

## Types of software needed for preparation and editing scientific manuscripts

Following software types are required to produce a publication-ready scientific manuscript (also see Table 1):

**Table 1:** List of software types, with commonly used proprietary software in Windows and available alternatives for Linux

List of Software	Most used in Windows	Alternatives for Linux
**Word Processor**	Microsoft Word	OpenOffice.org Writer LibreOffice Writer

**Spreadsheet**	Microsoft Excel	OpenOffice.org Spreadsheet LibreOffice Calc

**Presentation software**	Microsoft PowerPoint	OpenOffice.org Presentation LibreOffice Impress

**Statistical software**	SPSS Matlab SAS Splus S	R Front-ends of R

**Raster graphics editor**	Adobe Photoshop	GNU Image Manipulation Program

**Vector graphics editor**	Corel Draw	Inkscape

**Video Editor**	Various	Mplayer/Mencoder Front-ends of Mplayer/Mencoder

**Reference Software**	Thomson EndNote	Bibus Zotero

### Word processor

A fully functional word processor is a mainstay for manuscript preparation. The most commonly used program for this purpose remains Microsoft Word (Microsoft Corporation, Redmond, Washington) largely due to its widely used proprietary format Word Document (DOC), nowadays the most often requested format for manuscript submissions. Microsoft Word is natively available only for Windows and Mac operating systems. On Linux operating systems, it can be installed as Microsoft Office for Windows if special compatibility layers are used, like WINE [11] (Wine Is Not an Emulator, a recursive backronym - developed online) or CrossOver Impersonator [12] (Codeweavers, Saint Paul, Minnesota). Linux natively supports OpenOffice.org [13] (Oracle Corporation, Redwood Shores, California) and its derivative LibreOffice [14] (The Document Foundation, an online organization). Both are compete, full office suites and include word processors capable to produce complex text documents with tables and graphs. Like Microsoft Office, OpenOffice.org and LibreOffice suites also include other components from the office family similar to Microsoft PowerPoint and Microsoft Excel. In addition, there are smaller (and often less powerful) standalone open source programs which are not part of specific office application suites.

Writers of OpenOffice.org and LibreOffice can can read and save files in DOC format (however, some formatting alterations can be observed if opened with Microsoft Word). Other common formats, like for example RTF, are also supported. Since PDF format is now an open standard, open-source text editors can natively export documents as PDF. The native, open standard format for open source text editors is ODT which unfortunately is not widely accepted by the journals.

It should be mentioned that newer versions of Microsoft Office (2007 and above) also support PDF and ODT formats. According to the Microsoft Office website, some formatting issues may occur when opening ODT files [15]. Other open standard formats supported by Microsoft Office are Webpage (HTML), Rich Text Format (RTF) and plain text (TXT). DOCX (Word Microsoft Office Open XML Format Document) is a native format for Microsoft Office 2007 and newer versions. Similar to ODT, DOCX is an Extensible Markup Language (XML) - based file format, but unlike ODT it is not a true open standard yet [16].

A notably popular open standard formats appears to be TeX. TeX, a typesetting program, along with TeX-derived macro language LaTeX, is widely accepted in basic science journals (Table 2). It is especially useful for displaying complex mathematical formulas, hence its popularity in mathematics, statistics, physics, etc. There are several text editors for TeX/LaTeX [17]. Most common TeX/LaTeX output formats are TEX, DVI, and PDF. Of note, ODT format can be converted to TeX using an open source converter [18].

**Table 2:** Accepted manuscript submission formats by leading general science, biomedical, and clinical medical journals

Journal	Accepted formats for manuscript submission
New England Journal of Medicine [34]	PDF, DOC, WPD, TXT, RTF

Nature [29]	DOC, TEX**

Cell [37]	DOC, RTF, TXT

The Lancet [38]	DOC

Science [30]	DOC, TEX**, RTF*

JAMA [39]	DOC, WPD

The Journal of Biological Chemistry [35]	PDF***

Circulation [40]	DOC, WPD

PloS ONE [31]	DOC, TEX, RTF

PNAS [32]	DOC, RTF, TEX

BMC Journals [33]	DOC, RTF, TEX

* Some restrictions apply

** TEX files must be accompanied by a PDF version of the same text for visual reference

*** Although manuscripts must be submitted in PDF format, Microsoft Word is recommended to prepare the manuscript text

### Statistical software

Statistical software is necessary to perform data analysis and visualization, which is an important part of creating a manuscript. SPSS (Statistical Package for the Social Sciences - IBM, Armonk, New York), SAS (Statistical Analysis System - SAS Institute Inc, Cary, North Carolina), Matlab (Matrix Laboratory - MathWorks Inc, Natick, Massachusetts) are among the most widely used proprietary statistical programs. In an open-source world, a most widely used statistical software is R: A language and environment for statistical computing [19] (R Foundation for Statistical Computing, Vienna, Austria). R is capable of producing most complex calculations as well as some of the most sophisticated diagrams. It is a command-line program and has a steep learning curve which can be considered as a downside by some users. Fortunately, there are several graphical front-ends for R providing significant part of its functionality in a graphical environment [20].

### Graphical editor

Nowadays, post-processing of images acquired by cameras, microscopes, and various diagnostic equipment is becoming increasingly important. The most widely accepted raster graphics editor used by the scientists appears to be Adobe PhotoShop (Adobe Systems, San Jose, California), possessing all required functionalities for preparing publication quality images. An open source raster graphics editor that also meets these requirements is GNU Image Manipulation Program [21] (GIMP - developed online). GIMP's functionality can be further extended with various plugins [22].

Sometimes, there may be a need for a vector drawing software, like Corel Draw (Corel Corporation, Ottawa, Ontario). A fully functional open source vector graphics editor is Inkscape [23] (developed online), natively supported on Linux.

### Video editor

Motion images are commonly used to supplements the manuscripts. As a result, video editors are needed to edit (shorten, cut, crop, etc.) original video files, or create a motion image from image series (like DICOM - Digital Imaging and Communications in Medicine - a standard format for medical imaging). For Windows operating system, various free and non-free software is available that can perform some or all of these tasks. In Linux, an open source video encoding, decoding, and filtering tool called Mencoder [24] (developed online) offers all of these these functions. Mencoder is a companion software of a powerful media player Mplayer [25]. Both Mplayer and Mencoder are command-line tools, however various graphical front-ends also exist [26].

### Reference management software

Last but not least, "a must have" program for almost every scientist is a reference management software capable of reference searching and downloading from various databases (like MEDLINE in case of biomedical sciences). Another important requirement is an easy integration into the word processor of choice. The most widely used reference management software for Windows operating system is EndNote (Thomson Reuters, New York City, New York), which seamlessly integrates with Microsoft Word. An acceptable reference management software in Linux should not only meet these requirements, but it should also have the ability to share the databases with EndNote users. Currently, there are two such open-source programs available: Bibus [27] (developed online) and Zotero [28] (Center for History and New Media at George Mason University, Fairfax, Virginia). Both can integrate with OpenOffice, LibreOffice, as well as with Microsoft Office. Zotero is an add-on to the web-browser Mozilla Firefox (Mozilla Corporation, Mountain View, California) and thus also provides additional web-browser integration.

Table 1 summarizes the program categories with specific examples discussed above.

## Required file formats by journals for manuscript submission

Required formats of the submitted manuscripts were examined for 11 leading general science, biomedical, and clinical medical journals (Table 2). It appears that DOC is the most universally accepted file format for manuscript submission. Notably, almost every journal does accept alternative formats. As we see, this often includes TeX/LaTeX [29-33], a widely accepted open standard. Some journals accept the manuscripts in PDF [34,35], which also is an open standard file format. ODT, a standard format for open source word processors, was not found to be accepted by any of the journals examined.

## Conclusion

This manuscript was prepared using OpenOffice.org 3.3.1 with Zotero 2.0.9 extension for Firefox 3.6.15, and Zotero OpenOffice Integration Plugin 3.0b3. Ubuntu Linux 9.10 - Karmic Koala (Canonical Ltd, Douglas, Isle of Man) was used as an operating system. We were able to demonstrate that preparation and editing of a scientific manuscript in Linux is, in fact, possible. This manuscript was prepared as a DOC file (Microsoft Word 97/2000/XP format) since it appears to be the most widely accepted format by the journals in various fields (from basic science to clinical).

## Discussion

Preparation of this manuscript was quite easy since it did not involve use of software other than a word processor and a reference management program. Despite this, it proves the concept that Linux-based open source software can serve as a reasonable alternative to widely accepted proprietary programs for creating scientific manuscripts.

No cost, freedom from restrictions, and customizability, among others, are the advantages we start to appreciate when using the open source software. Despite this, switching from one program to another (especially replacing an entire operating system) can be related to many problems and inconveniences. The learning curve is not always as fast as we would like it to be. This may impact the productivity, and become a source of frustration. However, it has to be mentioned that none of this is inherent to Linux or, more generally, an open source software - we think that if used from the beginning as a primary environment (instead of switched to it), the issues discussed above should largely not be encountered. In addition, most of the open source programs mentioned above are cross-platform, thus compatible with Windows and/or Mac. This means that individual programs can be substituted as needed. In fact, there is a long spectrum to chose from, and readers should not think of it as a dichotomy of using proprietary software only vs. becoming completely "open source".

When creating the files in proprietary formats (like Word Document), some incompatibility still persists with the "gold standard" proprietary software [36]. We consider this a disadvantage, however this is not inherent either to Linux or to a specific open source program. These issues with incompatibility should decrease and ultimately disappear with more widespread use of open source software.

## Competing interests

The authors declare that they have no competing interests. Publication costs were covered by the German Research Foundation. Authors have no other funding to disclose.

## Authors' contributions

VT conceived the report, performed literature and internet search, performed key steps of this report on his personal computer. JDS was involved in drafting the manuscript and is responsible revising it critically. Both authors have read and approve the final manuscript.
